# Effects of different returning method combined with decomposer on decomposition of organic components of straw and soil fertility

**DOI:** 10.1038/s41598-021-95015-5

**Published:** 2021-07-29

**Authors:** Xiao Wang, Xuexin Wang, Peng Geng, Qian Yang, Kun Chen, Ning Liu, Yueling Fan, Xiumei Zhan, Xiaori Han

**Affiliations:** 1 College of Land and Environment, Shenyang Agricultural University, Shenyang, 110866 China; 2 Institute of Applied Ecology, Chinese Academy of Sciences, Shenyang, 110016 China

**Keywords:** Plant sciences, Ecology, Environmental sciences

## Abstract

In view of the problems of low straw decomposition rates and reduced soil fertility in southern Liaoning, China, we investigated the effects of no-tillage mode (NT), deep loosening + deep rotary tillage mode (PT), rotary tillage mode (RT) and the addition of decomposing agent (the next is called a decomposer) (NT + S, PT + S, RT + S) on the decomposition proportion of straw, respectively, by using the nylon net bag method in combination with 365-day field plot experiments. The decomposition rules of cellulose, hemicellulose and lignin as well as the dynamics of soil organic carbon (SOC), soil microbial biomass carbon (MBC) and soil dissolved organic carbon (DOC) in straw returned to the field for 15, 35, 55, 75, 95, 145 and 365 days were analyzed. The results showed that in the short term, the decomposition of straw was better in both the rotray tillage and deep loosening + deep rotary modes than in the no-tillage mode, and the addition of decomposer significantly promoted the decomposition of straw and the release of carbon from straw, among them, the RT + S treatment had the highest straw decomposition proportion and carbon release proportion in all sampling periods. After a one year experimental cycle, the RT + S treatment showed the highest proportion of cellulose, hemicellulose and lignin decomposition with 35.49%, 84.23% and 85.50%, respectively, and soil SOC, MBC and DOC contents were also higher than the other treatments with an increase of 2.30 g kg^−1^, 14.22 mg kg^−1^ and 25.10 mg kg^−1^, respectively, compared to the pre-experimental soil. Our results show that in the short term, to accelerate the decomposition rate of returned straw and increase the content of various forms of carbon in soil, rotary tillage can be used to return the straw to the field, while also spraying straw decomposer on its surface. This experiment used a new straw decomposer rich in a variety of microorganisms, combined with the comparison of a variety of straw return modes, and in-depth study of straw decomposition effects of cellulose, hemicellulose and lignin. Thus, a scheme that can effectively improve the decomposition rate of straw and the content of various forms of organic carbon in soil within a short period of time was explored to provide theoretical support for the southern Liaoning.

## Introduction

The total amount of straw in China is large, and the annual production of various types of crop straw has exceeded 900 million tons^1^. Currently, straw in many areas cannot be used rationally and is burned or discarded, it burning has many adverse effects on soil and air quality and poses a major threat to the environment^2,3^. Straw is an important biomass resource and should be used wisely^4^. In agricultural production, straw returned to cultivated land can improve soil fertility, increase the accumulation of soil organic matter and enhance soil quality^5–7^. However, some studies have confirmed that there are some problems in the process of returning the full amount of straw to the field. The large amount of straw accumulation can easily cause the imbalance of soil carbon and nitrogen ratio in the short term, and the excessive amount of un-decomposed straw remaining, which affects the sowing and emengence rate of the next crop^8^. In this experimental area, the above problem exists, and the straw abandonment rate is high in southern Liaoning because of the slow decomposition of straw. Years of straw burning and abandonment have not only polluted the environment, but also led to a decline in soil fertility and high fertilizer inputs.

We noted that different methods of returning straw to the field affected the decomposition rate of straw^9,10^. Presently, mulching and burying are the two main ways to return straw to the field^11^. In the straw mulch treatment, the straw is evenly spread on the soil surface, and in the straw burial treatment, it is fully mixed with the soil or directly buried in soil^12,13^. Different return patterns expose straw to different temperatures, moisture, microbial activity and soil porosity^14–17^. Among them, the effects of temperature and moisture on straw decay were also mainly manifested in the microbial populations and microbial activity^18^. Microorganisms are known to play a crucial role in straw decomposition and soil nutrient cycling, and higher straw decomposition rates are attributed to greater amounts of microorganisms^19,20^. To accelerate straw decay and ensure normal production of the next crop, we chose to apply a straw decomposer rich in microorganisms^21,22^. Straw decomposer is a microbial preparation composed of a variety of microbial communities that can accelerate degradation through the decomposition metabolism of fungi, bacteria and actinomycetes, breaking down cellulose hemicellulose and lignin into small molecule organic compounds and minerals^23,24^. This decomposer can prevent or reduce the adverse effects of excessive straw retention on crop growth, thus stabilizing and improving soil nutrient content and significantly increasing the rate of straw decomposition^25^.

Presently, there are many reports on the effects of tillage methods on straw decomposition and soil fertility^26–28^, but fewer studies on straw decomposition characteristics, carbon release patterns and effectiveness on soil organic carbon on the interaction of different return patterns and straw decomposers, further studies investigating the relationship between the proportion of cellulose, hemicellulose and lignin decay and the proportion of straw decay are even less. There is also a lack of literature to support the implementation and development of straw return initiatives in southern Liaoning. Our experiment aims to solve the problem of declining soil fertility and slow decomposition of straw in a short period of time in southern Liaoning, which affects the sowing and emergence rate of next season's crop. Here, we hypothesized that the decomposition proportion of organic components of straw and the proportion of straw carbon release are higher in the deep loosening + deep rotary tillage or rotary tillage mode than in the no-tillage mode, and the enhancement of soil carbon content is also higher than in the no-tillage mode during the one-year test cycle. The addition of straw decomposer may accelerate the decomposition of straw and its organic components, and promote the accumulation of various forms of organic carbon in the soil, so we predicted that deep loosening + deep rotary tillage mode combined with decomposer or rotary tillage mode combined with decomposer is the best way to improve the decomposition proportion of straw and the content of various forms of organic carbon in the soil. In order to test these hypotheses, we investigated (1) the effects of return methods and decomposer on the decomposition characteristics of straw and its organic fractions, (2) the effects of return methods and decomposer on the dynamics of soil organic carbon in different forms, and (3) the regulation of straw decomposition patterns.

## Materials and methods

### Site description

**Figure 1:**
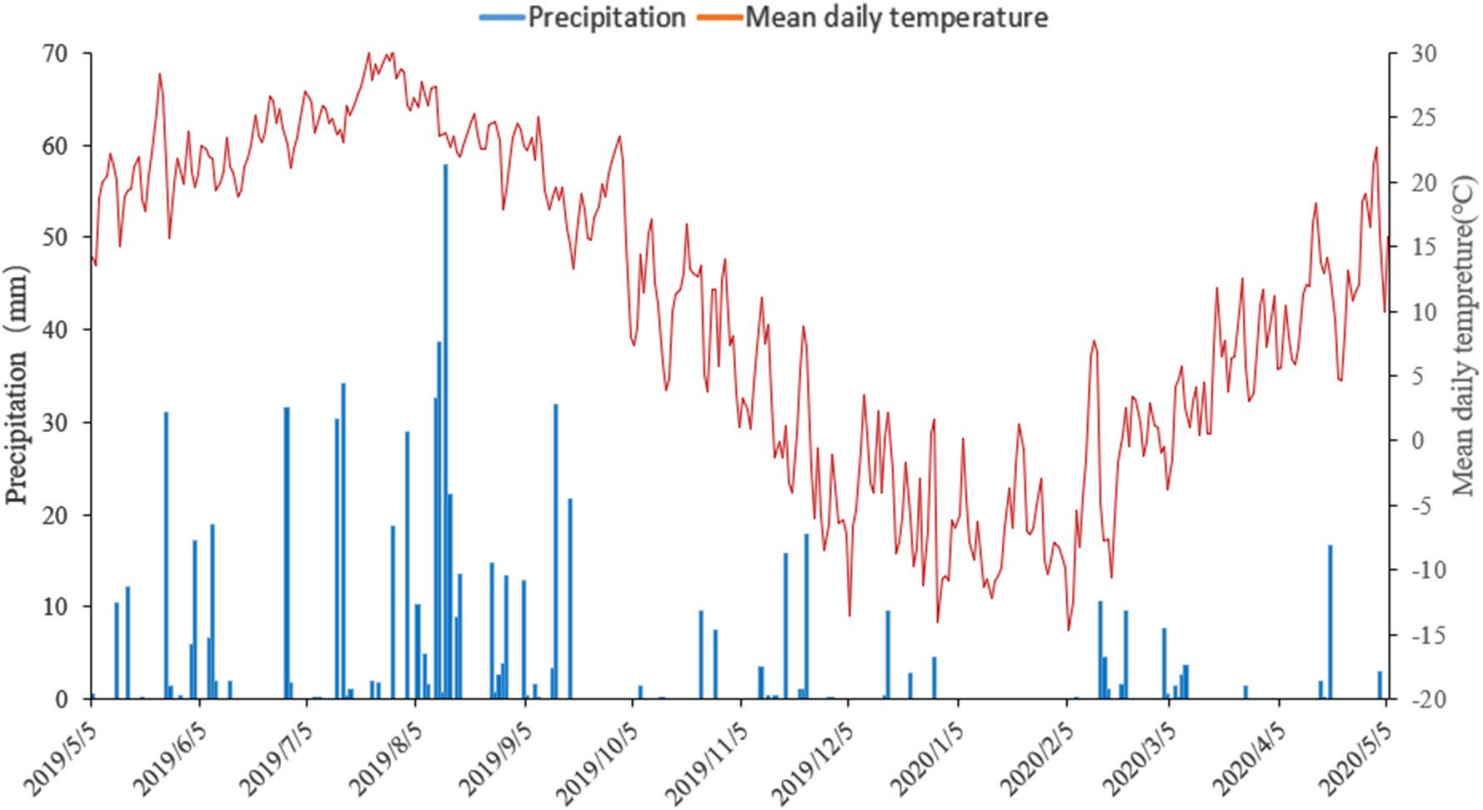
Daily precipitation and mean air temperature during the straw decomposition period from May 2019 to May 2020.

The experimental was conducted in Gengzhuang Town, Haicheng(40° 48′ N, 122° 37′ E), Liaoning Province from 2019 to 2020. This area is belonged to the continental monsoon climate zone of warm temperate zone, the annual average temperature is above 10 °C, the annual accumulated temperature is 3000–3100 °C, the frost-free period is about 170 days, and the annual rainfall is 600–800 mm. The soil at the experimental site is classified as brown earth^29^. Before the experiment, this test field had been in rotary tillage mode each year, with no straw return. The concentration of soil organic carbon, total nitrogen, available nitrogen, available phosphorus, available potassium, and soil bulk density in 0–20 cm surface layer were 12.50 g kg^−1^, 0.89 g kg^−1^, 129.6 mg kg^−1^, 25.96 mg kg^−1^, 117.94 mg kg^−1^, and 1.53 g cm^−3^, respectively. The components and nutrient contents of corn straw were shown in Table 1, The average temperature and precipitation from May 2019 to May 2020 are shown in Fig. 1.

**Table 1:** Initial component content of corn straw.

Cellulose %	Hemicellulose %	Lignin %	C %	N %	P %	K %
32.14	34.37	10.25	36.90	0.92	0.22	0.86

### Experimental design and management

We adopted a split plot design, with the main plot as the cultivation method, and with three cultivation options: no-tillage, deep loosening + deep rotary tillage and rotary tillage. Then, when adding straw as a decomposer, two methods were used: adding straw decomposer and not adding it. Our experimental approach included six treatments: No-tillage and straw mulching to the field + straw decomposer (NT + S); no-tillage and straw mulching to the field + no straw decomposer (NT); rotary tillage and straw mixed into the soil + straw decomposer (RT + S); rotary tillage and straw mixed into the soil + no straw decomposer (RT); deep loosening + deep rotary tillage and straw return to the field + straw decomposer (PT + S); and deep loosening + deep rotary tillage and straw return to the field + no straw decomposer (PT). Each treatment was replicated 3 times, located in random blocks, with a total plot area of 68.4 m^2^. At the same time as the previous year's corn harvest, straw was returned to the field, crushed to about 10 cm long, spread evenly on the ground. No-tillage mulching and straw return to the field is the direct no-tillage maize sowing operation in spring; In the deep loosening + deep rotary tillage treatment, the soil is turned using a subsoiler to a depth of 35 cm, and then the straw is mixed into the soil through deep rotary tillage (a depth of 30 cm); and rotary tillage involves mixing straw into the soil with a rotary tiller to a depth of 20 cm and then raking it flat.

Using the nylon net bag method (mesh bags were 5 cm × 6 cm, small; 15 cm × 20 cm, medium; and large, 25 cm × 35 cm; each size with an aperture of 100 mesh), we simulated three return modes. Soil added to the net bags was taken from the top 0–20 cm prior to sowing in 2019, in the corresponding plots of each treatment. Corn stalks were added at a ratio of 5:4 per stem and leaf (dry weight of stem and leaf of corn stalks in mature stage), and crushed to 2 cm long. In no-tillage treatment, 10 g straw was added to the medium mesh bag, in the rotary tillage and deep loosening + deep rotary tillage treatment, 10 g straw was evenly divided into five parts and put into five small net bags, then the five small net bags were evenly mixed into the soil of the outer large net bag and sealed, the weight of soil added to each large net bag is 2 kg, and the compactness between the inner net bag and the soil in the outer net bag was adjusted.

Net bag layout was determined according to different treatment tillage patterns, and bags were placed in the field on seeding day in 2019. Deep loosening + deep rotary tillage was achieved by ploughing furrows 30 cm long, 15 cm wide, and 35 cm deep between corn rows in corresponding plots, large net bags were buried vertically in the furrows, filled with soil and compacted, so that return depth and straw distribution were basically the same as deep loosening + deep rotary tillage in the field. Rotary tillage mode was achieved by ploughing furrows 30 cm long, 15 cm wide, and 20 cm deep between corn rows in the corresponding treatment plot, the packed net bags were tilted in the furrows, filled with soil and moderately compacted, the top end of the net bags was level with the ground surface, which is basically consistent with the return depth of rotary tillage and straw distribution in actual field production. No-tillage mulching treatments involved laying the net bags containing straw on the ground and covering the four corners with soil to prevent the net bag from being blown away by the wind. The decomposer addition treatments involved evenly spraying c. 6.5 ml straw decomposer on the straw surface before bagging, in the treatment without decomposer, 6.5 ml water was sprayed on the surface of straw to maintain the same water content.

In all treatments we applied the same amount of N, P and K (N 240 kg hm^−2^, P_2_O_5_ 74 kg hm^−2^ and K_2_O 89 kg hm^−2^). The nitrogen fertilizer was urea, the phosphate fertilizer superphosphate, and the potassium fertilizer, potassium chloride. The brand of straw decomposing agent is Gainby and the model number is d-68 (created by NORDOX company and produced by Beijing Shifang Biotechnology Co., Ltd.). Straw decomposer dosage was 1.5 kg hm^−2^, diluted with water 100 times, and the effective viable bacteria number was ≥ 50 million g^−1^. The effective bacteria in the decomposer include: Bacillus licheniformis, Aspergillus niger and Saccharomyces cerevisiae and so on.

### Sampling and analysis methods

On the 15th, 35th, 55th, 75th, 95th, 145th and 365th day after the nylon net bags were placed in the field plots, 3 bags were randomly sampled from each plot. For each net bag, we first washed the surface soil off with tap water, then washed the sample with distilled water 3 times, dried it at 60 °C, weighed it and then ground it to deter-mine the decomposition rate of straw and its components. At the same time, in the no-tillage treatments, 200 g soil was taken from 0 to 5 cm below the straw net bag, in rotary tillage and deep loosening + deep rotary tillage treatments, 200 g soil from net bag was taken for the determination of soil SOC, MBC and DOC. Content of cellulose, hemicellulose and lignin in straw were determined following Van’s method^30^, using a SLQ-6A semi-automatic crude fiber analyzer (Shanghai Fiber Testing Instrument Co., Ltd.).

The following formula was used to calculate decomposition rate of straw and its components. *M*_0_ is the initial straw or cellulose (hemicellulose, lignin) mass, g, and *M*_*t*_ is the straw or cellulose (hemicellulose, lignin) mass at time t, g.

1 $$\mathrm{Decomposition \; proportion }\left({\%}\right)= \frac{{M}_{ 0}-{ M}_{ t}}{{M \, }_{0}}\times 100.$$

The following formula was used to calculate the straw carbon release proportion. *C*_0_ is the initial straw carbon content, g, *C*_*t*_ is the straw carbon content at time t, g.

2 $$\mathrm{Straw \; carbon \; release \; proportion }\left({\%}\right)= \frac{{C}_{ 0} -{ C }_{t}}{{C }_{0}}\times 100.$$

The following formula was used to calculate the straw and its components decomposition rate. *M*_365_ is the quality of straw or cellulose (hemicellulose, lignin) mass on the 365th day, mg day^−1^.

3 $$\mathrm{Decomposition \; rate }\left(\mathrm{mg }{\mathrm{day}}^{-1}\right)= \frac{{M}_{ 0 }- {M}_{365}}{365}.$$

The relationship of the straw decomposition proportion (%) changes over time was fitted as follows:

4 $${y}_{t} = a+b\times exp \left(-kt\right),$$

where *y*_*t*_ is the proportion of the straw decomposition proportion at time *t*, %; t is the decomposition time of straw; *k* is the decomposition rate constant calculated using the least-squares method; *a* and* b* are constants.

SOC concentrations (g kg^−1^) was determined using the K_2_Cr_2_O_7_–H_2_SO_4_ digestion method^31^. Soil MBC content was determined using the Chloroform fumigation extraction method^32^. Two fresh soil samples were weighed, and then one of them was placed in a vacuum dryer with chloroform added, and pumped until the chloroform boiled violently, and after a period of time, the dryer cover was opened, the container containing chloroform removed, and the lid replaced. Another portion of soil was placed in a vacuum dryer without chloroform as a control. Then, 20 g each of fumigated and unfumigated soil samples were weighed, 50 mL 0.5 mg L^−1^ K_2_SO_4_ was added, extracted by vibration for 0.5 h, filtrate was pumped by 0.45 μm organic filter membrane, and then the filtrate was directly analyzed and detected using a TOC organic carbon analyzer. Based on the difference of organic C content between fumigated and unfumigated soil extracts, the microbial biomass carbon was obtained by multiplying the coefficient by 2.64. For the determination of soil DOC content, we used a slightly modified method of Jones^33^ and Hu Haiqing^34^. We made a leaching solution with 0.5 mol L K_2_SO_4_, weighed 10 g over 2 mm sieve of air dried soil, added the soil to the leaching solution to create a soil mass ratio of 2.5:1, and then applied a shock temperature for 1 h (220 r min^−1^). Then, after filtering, the filtrate was centrifuged for 20 min (3800 r min^−1^), filtered with a 0.45 μm organic membrane, and the filtrate subjected to TOC organic carbon analysis meter tests.

### Data analysis

In this experiment, Excel 2016 (Microsoft Corporation, New Mexico, USA) software was used to collate and analyze the data, and SPSS 19.0 (SPSS Inc., Chicago, Illinois, USA) statistical software was used to conduct variance analysis, LSD multiple analysis comparison and nonlinear regression analysis on the data. Duncan’s multiple range test was used to compare the treatment means at a 95% confidence level. Graphs were drawn using Origin 9.0 (Originlab, Northampton, USA).

## Results

### Decomposition of straw and its components

**Figure 2:**
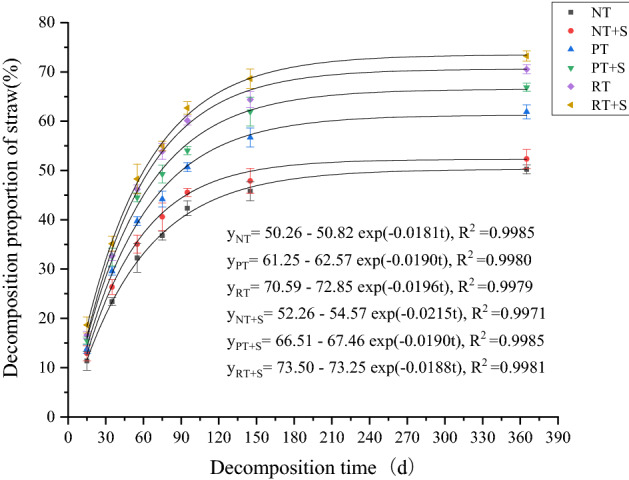
Fitting straw decomposition proportion curve by using the first order dynamic equation. *NT* no-tillage and straw return to the field + no straw decomposer, *NT + S* no-tillage and straw return to the field + straw decomposer, *PT* deep loosening + deep rotary tillage + no straw decomposer, *PT + S* deep loosening + deep rotary tillage + straw decomposer, *RT* rotary tillage + no straw decomposer, *RT + S* rotary tillage + straw decomposer. The same below.

From May to September 2019, the temperature and rainfall were higher, than from September 2019 to May 2020 (Fig. 1), and under the influence of temperature and moisture, the straw decomposition proportion in the early period was higher (0–95 days, 11.35–62.70%), while at the late stage it was lower (95–356 days, 42.35–73.25%). The decomposition proportion of straw and its organic components were higher for tillage than no-tillage mulching (Table 2). Moreover, decomposition proportion for rotary tillage was higher than deep loosening + deep rotary tillage. At 365 days, the straw decomposition proportion under no-tillage mulching was 50.25–52.35%, under deep loosening + deep rotary tillage 61.90–66.90%, and under rotary tillage 70.55–73.25% treatments, respectively (Fig. 2).

**Table 2:** Straw decomposition and carbon release rates under different return modes. F: Methods of returning farmland; Tr: processing. Different letters after the mean indicate a significant difference between treatments. *, ** and *** indicate a significance difference at 0.05, 0.01 and 0.001 levels, respectively. *NS* no significant difference.

Treatment	Tillage mode	Straw decomposition rate (mg day^−1^)	Cellulose decomposition rate (mg day^−1^)	Hemicellulose decomposition rate (mg day^−1^)	Lignin decomposition rate (mg day^−1^)	C releasing rate (mg day^−1^)
No decomposer	NT	13.8 ± 0.10 c	4.7 ± 0.10 b	6.0 ± 0.20 b	0.8 ± 0.01 c	3.6 ± 0.02 c
	PT	17.0 ± 0.04 b	6.5 ± 0.08 a	7.4 ± 0.20 a	0.9 ± 0.01 b	6.2 ± 0.05 b
	RT	19.3 ± 0.06 a	6.6 ± 0.10 a	7.5 ± 0.10 a	2.1 ± 0.03 a	6.9 ± 0.07 a
Decomposer	NT	14.4 ± 0.02 c	5.7 ± 0.20 b	6.6 ± 0.10 b	0.8 ± 0.02 c	5.17 ± 0.08 c
	PT	18.3 ± 0.30 b	7.4 ± 0.10 a	8.1 ± 0.20 a	1.0 ± 0.02 b	6.6 ± 0.10 b
	RT	20.1 ± 0.20 a	7.4 ± 0.03 a	8.1 ± 0.30 a	2.4 ± 0.07 a	7.17 ± 0.80 a
F Tr F × Tr		32.6***	4.1***	2.8***	2.6***	4.0***
		2.4***	2.2***	1.1**	0.04***	0.3***
		0.176*	0.01	0.009	0.009***	NS

The first-order kinetic curve was used to fit the straw decomposition decomposition line chart (Fig. 2), it was found that the fitting degree of each treatment was good. It took 291.5 days, 148 days, 90.3 days, 74 days, 64.5 days and 60.4 days for NT, NT + S, PT, PT + S, RT and RT + S to decompose the straw to 50%, respectively.

**Figure 3:**
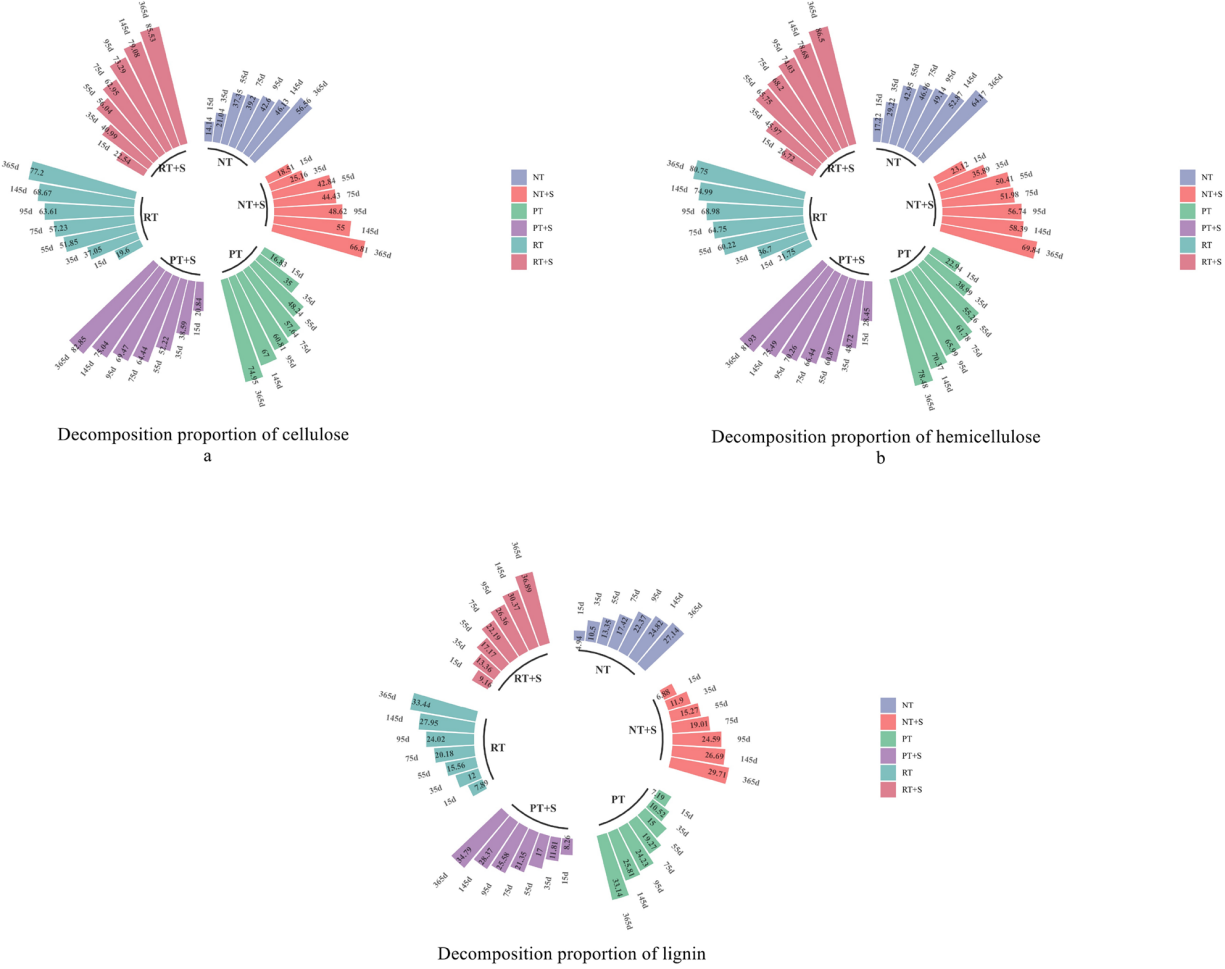
(**a**–**c**) are the circular barplots of the decomposition proportion of cellulose, hemicellulose and lignin in straw at 15, 35, 55, 75, 95, 145 and 365 days.

Under the same tillage method, the addition of decomposer promoted straw decomposition over the entire experiment, but its influence did not exceed the influence of tillage. The decomposition of cellulose, hemicellulose, and lignin in the straw was consistent with that of the straw, and straw decomposition proportion under deep turning + deep rotary tillage and rotary tillage was higher than that under no-tillage mulching. Under the same tillage pattern, the decomposition proportion of straw organic components treated with decomposition agent was significantly higher than that without it. At 365 days, the lignin decomposition proportion was lower than that of cellulose and hemicellulose. The decomposition proportion of lignin, cellulose and hemicellulose were 27.14–36.89%, 56.56–85.53% and 64.17–86.50%, respectively. The straw and cellulose and hemicellulose decomposition proportion under rotary tillage and deep loosening + deep rotary tillage were higher than those under no-tillage modes (Table 2, Fig. 3).

### Straw carbon release

**Figure 4:**
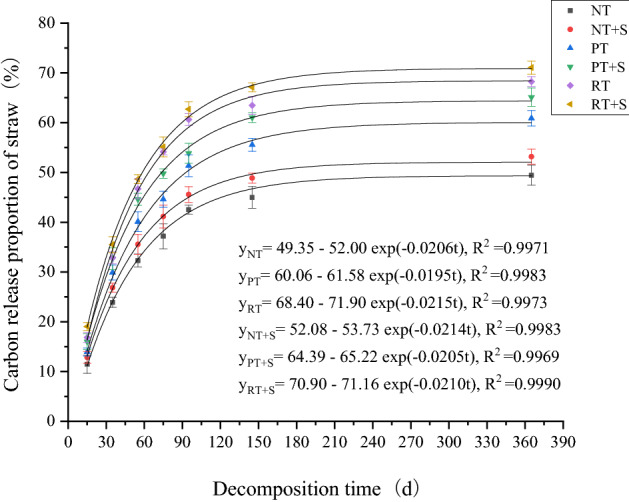
Fitting straw carbon release proportion curve by using the first order dynamic equation.

Trends in straw carbon release proportion and straw decomposition proportion were basically the same, being initially fast, and slow in later stages. Average carbon release proportion of straw under rotary tillage mode was higher than under the other two tillage treatments. Carbon release rates of straw between the three treatments was as follows: rotary tillage > deep loosening + deep rotary tillage > no-tillage (Table 2). At 365 days, the carbon release proportion of each treated straw was 49.44–71.05%. For the same return method, adding straw decomposition agent promoted carbon release. RT+S treatment had the highest proportion of straw carbon release in each time period among different return methods (Fig. 4).

First-order dynamics curve was used to fit the straw carbon release proportion line chart (Fig. 4). The time for NT + S, PT, PT + S, RT and RT + S treatments to release 50% of straw carbon was 152 days, 93 days, 73.7 days, 59 days and 58.8 days, respectively. It is worth mentioning that the straw carbon release proportion of NT treatment did not reach 50% within 365 days.

### Dynamic changes in soil organic carbon and soil microbial biomass carbon

**Figure 5:**
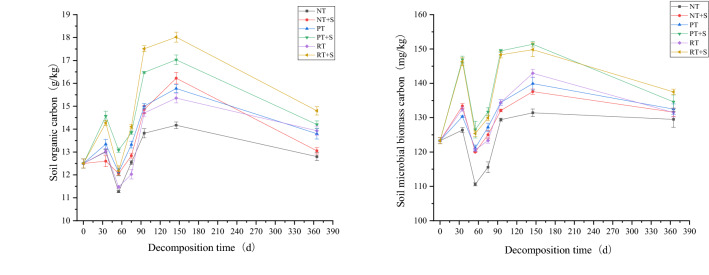
Changes of soil organic carbon and microbial biomass carbon under different returning modes.

From 0 to 35 days, the SOC content of each treatment was slightly elevated compared to the pre-test soil, PT + S and RT + S treatments improve more. From 35 to 55 days after straw return, SOC content gradually decreased in each treatment, reaching 11.27–12.13 g kg^−1^ at day 55. From day 55 to day 145, the change in SOC content was generally consistent with the proportion of straw carbon released, showing a rapid increase and reaching the maximum value for the year at day 145, followed by a decreasing trend in SOC content for all treatments until day 365. The SOC content of RT + S treatment was higher at day 145 (18.02 g kg^−1^) and day 365 (14.80 g kg^−1^) were higher than the other treatments. By the 365th day, for the same mode of straw return to the field, SOC content of the treatments with the addition of decomposer higher than without it, and the SOC contents of the four treatments PT + S, RT + S, RT and PT were significantly higher than the pre-experimental soil (Fig. 5).

During the entire experimental period, the changes in MBC content were largely consistent with the changes in SOC content. At day 145, the MBC contents of PT+S and RT+S were 151.38 mg kg^−1^, 149.82 mg kg^−1^, respectively. From 0 to 365 days, PT+S (365 days, 134.61 mg kg^−1^) and RT+S (365 days, 137.52 mg kg^−1^) treatments MBC content were always at a relatively high level and higher than other treatments, but there was no significant difference between the two treatments. NT treatment MBC content was lower than in other treatments. Under the same return method, MBC content in the treatments with straw decomposer were higher than those without it (Fig. 5).

### Changes in soil dissolved organic carbon

**Figure 6:**
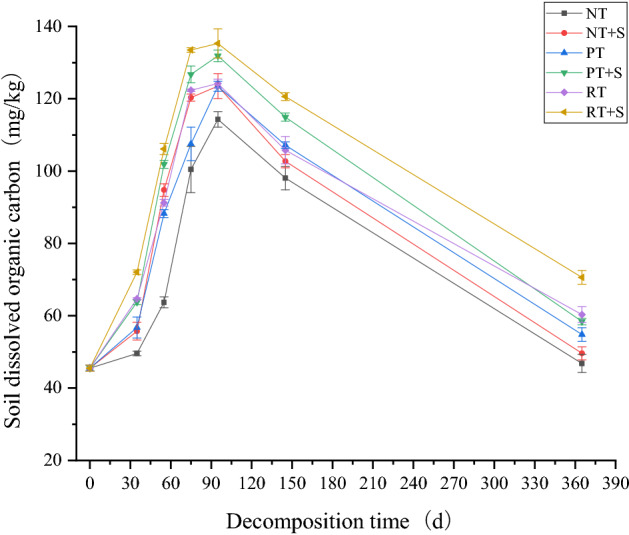
Changes of soil dissolved organic carbon under different returning modes.

Generally, both the straw return method and the decomposer had effects on DOC content: rotary tillage > deep loosening + deep rotary tillage > no-tillage; and straw decomposer addition > no straw decomposer. After straw was returned to the field, soil DOC content of all treatments increased rapidly with the rapid decomposition of straw and release of straw carbon, reaching a maximum of 114.29–135.29 mg kg^−1^ on the 95th day. From 95 to 365 days, soil DOC content of all treatments began to decrease gradually. During the 0–365 days, soil DOC content in RT + S and PT + S treatments was generally at a higher level, and RT + S was higher, than other treatments. NT treatment soil DOC content was always the lowest. After one year decomposition cycle, except for the two treatments in no-till mode, the DOC content of the other four treatments significantly increased compared to the pre-experimental soil (Fig. 6).

## Discussion

Several studies have shown that that higher temperature and suitable moisture can increase microbial activity and diversity, and the activity and abundance of soil microorganisms are crucial to the decomposition rate of straw and the increase of SOC content^35–37^. In our study, the NT and NT + S treatments showed greater temperature variation and the highest oxygen content, but the percentage of straw decomposition and the organic carbon content of each form were always low, probably due to the fact that under no-tillage mulching conditions, the straw was exposed to the surface and did not have sufficient contact with soil and water, and the decomposer was exposed to the ground, where microorganisms could not function because of sun exposure and lack of water, thus affecting the decomposition of straw and the release of nutrients^38,39^. Therefore, we believe that in the future no-tillage mulching straw return measures, we can choose to add straw decomposer or not depending on the situation, because the effect of decomposer is not obvious. On the other hand, the straw decomposition rate was significantly higher in the two modes of buried return than in the no-tillage mode, and all indicators were at a higher level in the RT + S treatment, which is consistent with the results of Lu et al.^27,40^. It might be because under the buried return condition, straw was mixed with soil and microorganisms were provided with suitable temperature and moisture conditions, and the addition of decomposer greatly increases the diversity and abundance of microorganisms^13,26^, thus significantly increased the decomposition proportion and carbon release proportion of straw. In deep loosening + deep rotary tillage treatments, part of the straw was about 30 cm below the soil surface, organic matter was in an anaerobic environment, and anaerobic conditions inhibited the activity of aerobic microorganisms^41^, under which the relative content of difficult-to-degrade straw components such as lignin increased, reducing straw degradation rate^42^. However, when rotary tillage straw return was shallow, total soil porosity was higher, and it was easier to access oxygen, so straw decomposition proportion was higher than deep loosening + deep rotary tillage^13^. The proportion of straw decomposition and its organic components increased rapidly in each treatment from 0 to 145 days after straw return. There is evidence that at the early stage of straw return, soluble carbohydrates, organic acids, amino acids and other non-structural easily decomposable substances are rapidly released, providing soil microorganisms with a large source of carbon and nutrients, thereby increasing their number and activity, and accelerating the decomposition of straw organic components^36^. Additionally, rainfall and temperature during this period gradually increased to their annual maximum, which can lead to increased microorganism activity and reproductive capacity, thus speeding up straw decomposition^43^. During the 145–365 day period, the temperature and precipitation gradually dropped, and after c. 200 days winter arrived, and the surface soil temperature was close to or less than zero, and microbial activity slowed down and gradually entered dormancy, so the proportion of straw decomposition gradually leveled off^44^.

To enhance soil fertility, one of the typical agricultural methods is to return straw to the field^45^. Microbial communities and microbial activity are the two main factors affecting MBC and SOC under straw return conditions^46^, and there is also evidence that the presence and abundance of microorganisms play an extremely important role in the dynamics of soil DOC^47^. Therefore, in this experiment, the addition of decomposer increased the content of soil SOC, MBC and DOC under the same tillage pattern, and this result is consistent with the results of Wang et al.^21^. In our experiments, compared to other treatments, the rotary tillage mode, which is more amenable to microbial survival, had a higher proportion of straw carbon release and higher SOC and DOC content, and the RT + S treatment was the highest, indicating that the rotary tillage mode combined with decomposer had a significant effect on straw carbon release and soil carbon enrichment during the 1-year decomposition cycle. Interestingly, the MBC contents of both RT + S and PT + S treatments were similar throughout the decomposition process, and the SOC and DOC contents of both treatments were also higher than the other treatments, which may be due to the relative similarity of both treatments (except for tillage depth). The addition of straw decomposer led to similar microbial amount of the two treatments, but in actual agricultural production, the mechanical cost input of deep loosening + deep rotary tillage is much higher than rotary tillage. Therefore, since rotary tillage mode is more energy efficient and environmentally friendly, it is more conducive to widespread use. The lowest SOC and MBC content for each treatment occurred on day 55, we speculate that this is because maize is at the peak of nitrogen uptake at the jointing stage, but soil microorganisms also need abundant nitrogen to meet their reproduction needs, so the soil C/N ratio increased significantly, inhibiting microbial reproduction and leading to a decrease in soil microbial biomass carbon content^48^. The sudden addition of exogenous organic matter stimulated the original organic matter in soil and promoted the decomposition of SOC, resulting in a decrease in SOC content at this stage^42,49^. According to the study, the activity of microorganisms that degrade the soil substrate increases due to the increase in temperature^50^, which leads to an exponential function increase in soil DOC and MBC content, and the increase in MBC may promote the decomposition of straw and the subsequent increase in soil SOC content^13^. Similar results were obtained in this experiment, where the organic carbon content of all soil forms increased rapidly with the increase in temperature from day 35 to 95 of straw return, after 145 days of straw return, the MBC and SOC contents decreased slowly as the straw decomposition became slow. On day 365, the order of SOC content of each treatment from highest to lowest was RT + S, PT + S, RT, PT, NT + S, and NT, indicating that the addition of decomposer significantly improved the accumulation of SOC, with RT + S having the best effect on soil fertility.

## Conclusion

Among the three return modes, the effects of rotary tillage and deep loosening + deep rotary tillage modes on straw decomposition and straw carbon release proportion were better than those of no-tillage mode, and this conclusion can also be verified by calculating the half-life of straw decomposition proportion and carbon release proportion through the fitting curve of the first-order dynamic equation. Among all treatments, the RT + S treatment had the highest proportion of straw decomposition and carbon release in each sampling period. At day 365, the RT + S treatment had the highest proportion of decomposition of cellulose, hemicellulose and lignin, and the soil SOC, MBC and DOC contents were also higher than the other treatments, the decomposition proportion of cellulose and hemicellulose was significantly higher than that of lignin, the decay of straw is mainly due to cellulose, hemicellulose decay, moreover, the decomposer can accelerate the decomposition of organic components of straw. In the short term, to accelerate the decomposition rate of returned straw and its organic fraction and to increase the content of various forms of carbon in soil, rotary tillage can be used to return the straw to the field, while spraying straw decomposer on the surface of returned straw. Our study highlights were: (1) we explored the effects of three different tillage modes (no-tillage, rotary tillage, and deep loosening + deep rotary tillage) combined with decomposers on straw decomposition and soil nutrients, whereas previous studies of straw decomposition have mostly focused on the effects of tillage methods or decomposer alone; (2) we further investigated the effect of microbial inputs on the decomposition of straw organic components (cellulose, hemicellulose, and lignin). And it was found that these three organic components affected the decomposition proportion of straw in different degrees; and (3) in recent years, soil fertility in southern Liaoning has been declining, as there has been a shortage of suitable straw return programs, and so our study provides a theoretical basis for implementing a scientific straw return program in this area.
